# Key Food Hygiene Behaviors to Reduce Microbial Contamination of Complementary Foods in Rural Bangladesh

**DOI:** 10.4269/ajtmh.21-0269

**Published:** 2022-07-25

**Authors:** Anna A. Müller-Hauser, Shafinaz Sobhan, Tarique Md. Nurul Huda, Jillian L. Waid, Amanda S. Wendt, Mohammad Aminul Islam, Mahbubur Rahman, Sabine Gabrysch

**Affiliations:** ^1^ Charité—Universitätsmedizin Berlin, corporate member of Freie Universität Berlin and Humboldt-Universität zu Berlin, Institute of Public Health, Berlin, Germany;; ^2^ Research Department 2, Potsdam Institute for Climate Impact Research (PIK), Member of the Leibniz Association, Potsdam, Germany;; ^3^ Heidelberg Institute of Global Health, Heidelberg University, Heidelberg, Germany;; ^4^ Environmental Interventions Unit, Infectious Diseases Division, International Centre for Diarrhoeal Disease Research, Bangladesh (icddr,b), Dhaka, Bangladesh;; ^5^ Paul G. Allen School for Global Health, Washington State University, Pullman, Washington;; ^6^ Food Microbiology Laboratory, Laboratory Sciences and Services Division, icddr,b, Dhaka, Bangladesh

## Abstract

Microbial contamination of complementary foods puts young children at risk of developing intestinal infections and could be reduced by improved handwashing and food hygiene practices. We aimed to identify which promoted food hygiene practices are associated with reduced complementary food contamination in a rural population in Bangladesh. We collected cross-sectional data on reported and observed maternal food hygiene behaviors and measured *Escherichia coli* counts as an indicator of microbial contamination in complementary food samples from 342 children of women enrolled in the Food and Agricultural Approaches to Reducing Malnutrition trial in Sylhet, Bangladesh. We used multivariable logistic regression to examine associations of food hygiene behaviors with food contamination. Approximately 46% of complementary food samples had detectable levels of *Escherichia coli*. Handwashing with soap at critical times and fresh preparation of food before feeding were strongly associated with reduced odds of food sample contamination (odds ratio [OR]: 0.8, 95% confidence interval [CI]: 0.6–0.9 and OR: 0.3, 95% CI: 0.1–0.7, respectively); in contrast, there was no or only weak evidence that reheating of stored food, safe food storage, and cleanliness of feeding utensils reduced contamination. Reduction in food contamination could be more than halved only when several food hygiene behaviors were practiced in combination. In conclusion, single food hygiene practices showed limited potential and a combined practice of multiple food hygiene behaviors may be needed to achieve a substantial reduction of complementary food contamination.

## INTRODUCTION

Intestinal infections and diarrhea are a global problem. Yearly, ∼550 million episodes of diarrhea are caused by consumption of food contaminated with foodborne pathogens, of which ∼40% occur in children under 5 years of age.^1^^,^^2^ In addition to diarrhea, exposure to contaminated food can cause a subclinical inflammatory disorder of the intestine, termed environmental enteric dysfunction, which involves an impaired barrier function of the intestinal lining.^3^ Young children are particularly vulnerable to intestinal infection and disease because their bodies cannot compensate for the lost energy and micronutrients and thereby maintain proper growth and development.^4^ As a result, high rates of diarrheal disease and intestinal infection increase the risk of malnutrition and stunting in young children.^5^^–^^7^

In resource-poor settings, complementary foods fed to young children are often highly contaminated with pathogenic microbes.^2^^,^^8^^–^^12^ There is evidence that suboptimal household hygiene is associated with contamination of complementary foods.^13^^,^^14^ Hazard analyses have helped to identify critical control points in the food preparation chain that could lead to a potential introduction of contamination, such as 1) use of contaminated foods from field and markets, 2) inadequate handwashing practice (especially before food preparation and feeding), 3) cleaning of cooking and feeding utensils with contaminated water, 4) a long lag time between food preparation and actual feeding, combined with unsafe storing practices at high ambient temperature, and 5) inadequate reheating practice.^9^^,^^12^^,^^15^^–^^21^ Therefore, good caregiver hygiene practices around food preparation and child feeding could be an important means to reduce complementary food contamination in the household setting.

The impact of food hygiene recommendations on the reduction in food contamination varies in relation to the ability of the behavior to reduce contamination and the uptake of the behavior in different local settings.^22^ In addition, environmental conditions (e.g., housing, climate, availability of necessary infrastructure) might render a food hygiene behavior harder to practice or less effective.^22^^,^^23^ It is therefore important to assess the impact of food hygiene behaviors within the local context to identify practices that work best to inform and prioritize suitable future intervention messages.

The aim of this study is to better understand the potential impact of improved food hygiene practices on complementary food contamination within the setting of a Homestead Food Production trial in rural Bangladesh. We identify which of the promoted food hygiene behaviors were associated with reduced complementary food contamination. In addition, we predict prevalence of complementary food contamination when multiple food hygiene behaviors were practiced and assess to what extent food contamination could be prevented if single food hygiene behaviors were practiced perfectly by the entire study population.

## METHODS

### Study population.

Data analyzed for this study are from the Food and Agricultural Approaches to Reducing Malnutrition (FAARM) cluster-randomized controlled trial and the Food Hygiene to reduce Environmental Enteric Dysfunction (FHEED) study conducted within FAARM.^24^ The study area is located in rural Habiganj district, Sylhet division, Bangladesh. The FAARM trial aims to evaluate the impact of a Homestead Food Production program, implemented by the international nongovernmental organization Helen Keller International, on undernutrition in young children. The intervention targeted young women of reproductive age and included trainings on year-round gardening, poultry rearing, and improved nutrition and hygiene practices.^24^ The FAARM trial enrolled 2,700 married women in 96 settlements (geographic clusters), randomized into 48 intervention and 48 control clusters. Further information about the FAARM trial design can be found in the study protocol.^24^

To strengthen the food hygiene aspect of FAARM, an additional behavior change component was implemented and delivered to the intervention households from July 2017 to February 2018, promoting four hygiene behaviors around food preparation and child feeding.^25^ The four promoted behaviors covered 1) handwashing with soap and water before cooking, child feeding, and/or eating; 2) washing utensils with soap and water before preparing and serving food; 3) safe storage of cooked food and drinking water; and 4) cooking food fresh or thorough reheating of stored food before feeding/eating.

The analysis presented here is based on a subsample of 342 FAARM households that had a child aged 7 to 18 months at the time of food sampling (i.e., within the first year of complementary feeding, if the WHO recommendation of exclusive breastfeeding for the first 6 months of age was followed). As practicing food hygiene behaviors is not conditional on receiving a hygiene intervention, we included both intervention and control households to maximize power for identifying food hygiene behaviors associated with food contamination.

### Data collection.

For this analysis, we use data from three datasets: 1) background characteristics at the time of the FAARM baseline survey, 2) reported food hygiene behaviors collected as part of the FAARM’s routine assessment surveillance, and 3) direct observation of behaviors and collection of food samples. The FAARM baseline survey was conducted in 2015 and collected data on household and woman characteristics, such as age, education, household wealth, and religion from all households.

The FAARM surveillance system’s routine assessment was conducted on a bimonthly basis from 2015 to 2019.^24^ Data on reported food hygiene behavior were collected through a module added to the surveillance for two consecutive rounds from December 2018 to March 2019, after the conclusion of all food hygiene trainings. The food hygiene module targeted all households with a child under 18 months of age. Questionnaires were administered by trained data collection officers, conducting face-to-face interviews with the respondents. To minimize bias, questions on food hygiene practices were asked in an open, nonleading way, announcing to participants that these were about activities they might have done around the house. Whenever possible, data on hygiene behaviors were collected through spot-checks (e.g., to learn about food storage practices, participants were asked to show the food storage area to the interviewer).

During a separate cross-sectional survey from July to September 2018 for FHEED, we did household spot-checks around sanitation, kitchen, and food storage facilities, along with complementary food sampling, in all households with a child aged 7 to 18 months. Trained observers performed structured observations of household food hygiene behaviors over 3 hours, either in the morning or early afternoon. Observations focused especially on caretaker practices around complementary food preparation and child feeding, as well as handwashing behavior. To minimize bias, attendants were told that the observations were conducted to learn about daily household activities. All survey data were gathered using tablet-based Open Data Kit (ODK) software.^26^

### Microbiological sample collection.

As part of the cross-sectional survey, food samples were either sampled just before child feeding, or, if no feeding event was observed (in 19% of households), mothers were asked to prepare and serve food as if they would serve it to their 7- to 18-month-old child. Most complementary foods had multiple components, usually mixed before feeding, which were sampled together. Before food collection, temperature of the prepared food was measured using a food thermometer (SveBake, Model TP500, China). Mothers were asked to place the food sample into a sterile plastic bag, which was then immediately stored in an ice-cooled bag and transported to icddrb’s food microbiology laboratory within 12 hours after collection, maintaining a cold chain (8–10°C) at all times.

### Enumeration of *Escherichia coli.*

Food contamination was assessed by counting colony-forming units (CFU) of *E. coli*, a WHO-recommended indicator organism for measuring fecal contamination.^27^^,^^28^ For enumeration of *E. coli*, standard methodology was followed.^29^ Briefly, an aliquot of 25 g solid or 25 mL liquid food sample was mixed well with 225 mL of 0.1% peptone water and homogenized in a Stomacher 400 circulator (Seward Co. Ltd., West Sussex, UK) at 230 rpm for 1 minute. One milliliter of the suspension was transferred to a tube containing 9 mL of sterile diluents and mixed thoroughly to obtain 10^−2^ dilution. If necessary, further dilutions were made to obtain 10^−3^, 10^−4^, and so on, until the appropriate number of microorganisms was obtained. For each food sample, we inoculated two plates per dilution and incubated at 44°C for 18 to 24 hours. Appearance of blue-green colonies on the TBX plate was indicative of the presence of *E. coli* and reported as CFU per gram of food (CFU/g). Samples negative for colonies on the initial dilution plate (10^−1^) were reported as < 10 CFU/g. For samples with colonies too numerous to count, further dilutions were plated and incubated. We used *E. coli* 25922 as a positive control, *Klebsiella pneumoniae* FML 201 as a negative control, and 0.1% peptone water inoculated directly to the media by pour plating as media control.

### Variables.

Complementary food contamination was the outcome of interest. Two categorical variables were generated from our measure of the number of *E. coli* CFU. Food contamination was defined as binary variable, based on presence or absence of detectable *E. coli* in the food sample (*E. coli* contamination yes/no). Negative results were reported as < 10 CFU/g food because this was the limit of detection of the plating method. Food contamination was also assessed as categorical variable, *E. coli* contamination absent/low (< 10 CFU/g food), medium (10–100 CFU/g food), and high (> 100 CFU/g food), given that 100 CFU/g is set as the safety threshold for ready-to-eat foods in microbiological food quality guidelines.^30^ In addition, log-transformed *E. coli* counts were used as continuous outcome (log CFU/g food).

Using the surveillance data, we categorized reported caregiver food hygiene practices as binary variables in line with key food hygiene behaviors: 1) handwashing with soap was reported for five critical time points: i) before food preparation, ii) before child feeding, iii) after defecation, iv) after cleaning the child, and v) after handling animal feces. In addition, reported handwashing at critical times was summarized into a handwashing score, ranging from 0 to 5. 2) Child feeding utensils were considered clean when the caregiver reported washing with soap and water from a clean water source and storing at a clean place. 3) Storage of food and water was considered safe when the caregiver reported that food and water were fully covered and elevated from the ground. 4) Food was considered fresh or properly reheated when the caregiver reported fresh cooking or reheating of stored food until steaming hot.

From household spot-checks and observations, we created variables in line with the key food hygiene behaviors: 1) Handwashing was observed at the five critical time points as listed above, with acceptable handwashing practice defined as handwashing with soap and water, washing palms and back of both hands for at least 3 seconds, and drying hands either with a visibly clean cloth or letting them air-dry. If a respondent practiced a handwashing behavior multiple times, the behavior was only considered as acceptable handwashing practice when practiced well at all times. 2) Child feeding utensils were considered clean when they were either washed and stored at a clean place or washed with soap right before child feeding. In case a child feeding event was observed multiple times during the observation period, feeding utensils were only considered clean if clean utensils were used at all feeding events. When children were fed by hand, feeding utensils mainly comprised the plate or bowl. Cleanliness of hands, often directly used in feeding, was covered in the variable ‘handwashing before child feeding’. 3) Storage of food and water was considered safe when food and water were stored fully covered, elevated from the ground, and without animals visible in the storage area. 4) Sampled food was categorized into three groups: food prepared fresh, food that had been stored but reheated, or food that had been stored but not reheated.

The household environment was observed and categorized as follows: 1) a handwashing station was considered functional when it was at a fixed location and equipped with water, cleaning agent, and a pouring device (WHO and UNICEF Joint Monitoring Program (JMP) classification), and 2) the kitchen and food preparation area were considered clean when they were free from visible dirt, fecal contamination, and animals. In addition, the latrines used by the households were categorized according to WHO and UNICEF JMP classification. Because information on feces disposal was not available, the highest category (safely managed) was omitted, and latrines were classified as “open,” “unimproved,” “limited,” and “at least basic.”

Additional food characteristics that could influence food contamination were described and included in the analysis. We classified type of food as porridge, khichuri (jointly cooked rice and pulses), plain rice, two types of mixed rice dishes, and others. Food storage time was categorized with cutoffs at 2, 4, and 8 hours. The ambient temperature of the food storage area was measured in degree Celsius and relative humidity of the food storage area as a percentage of the maximum possible humidity given the same temperature. To adjust for other potential unmeasured effects caused by the intervention that could influence the relationships observed, we also included intervention arm as a potential confounder.

We considered maternal education and literacy, as well as household religion, household wealth, and the number of persons living in the household as further potential confounders of the relationship between food hygiene characteristics and food contamination. These indicators were all associated with the uptake of food hygiene behaviors in the evaluation of the food hygiene behavior change component.^25^ Data on these potential confounders were collected during the FAARM baseline survey in 2015. Education was measured as number of school years completed, and literacy was defined as the ability to read a simple sentence in Bangla. Household wealth quintiles were calculated using principal components analysis of a household asset list adapted from the Bangladesh Demographic and Health Survey.^31^ Characteristics showing an association (*P* < 0.1) with food contamination and with at least one of the exposure variables, which was the case for household wealth and maternal literacy, were included in multivariable models. In addition, we examined whether findings were altered by including all other potential confounders, which was not the case.

### Statistical analysis.

We performed all data analyses in Stata 14. We described outcome, exposure, and confounder variables using proportions or means and standard deviation. We used bivariable logistic regression to assess crude associations of single food hygiene characteristics with food contamination, adjusting for clustering at settlement level using random effects. To identify determinants of food contamination, we used mixed effects multivariable logistic regression models including all reported or observed food hygiene behaviors, accounting for clustering at settlement level and adjusting for type of food, food storage time, temperature and humidity of the food storage area, intervention allocation, maternal literacy, and household wealth as potential confounders. For *E. coli*--positive samples, we log-transformed *E. coli* counts and conducted mixed effects multivariable linear regression including all observed food hygiene behaviors, accounting for clustering at settlement level and adjusting for confounding in the same way. Variables in the final models were assessed for multicollinearity by calculating variance inflation factors, which were all close to 1. Predicted probabilities of food contamination were estimated and plotted by calculating marginal effects from the final logistic regression model, using the Stata commands *margins* and *marginsplot*. Population attributable fractions (PAF) were calculated following multivariable logistic regression, using the Stata command *punaf*. The command cannot be used after multilevel models, therefore these estimates were made using robust standard errors.

### Ethics.

The FAARM and FHEED study protocols were positively reviewed by ethics committees in Bangladesh and Germany, and written informed consent was obtained from all study participants before data collection.^24^

## RESULTS

### Sample characteristics.

Sociodemographic characteristics of the study population are summarized in Table 1. Overall, a little less than half of the 342 complementary food samples (46%) were contaminated with *E. coli*; 18% had a medium level of contamination (10–100 CFU/g food), and 28% were highly contaminated (> 100 CFU/g food; Table 2). The most common complementary foods were dishes of plain rice served with a variation of vegetables and/or lentils, egg, fish, or meat (71% of food samples; Table 2). Other foods commonly served were porridge (10% of complementary foods) and khichuri, a traditional dish of jointly cooked rice and pulses (18% of complementary foods). Approximately 40% of complementary foods had been stored for more than 4 hours (Table 2).

**Table 1:** Characteristics of participant women and households in Sylhet, Bangladesh

Characteristics	Frequency	%
Maternal age, years		
< 20	50	15
20–24	167	49
25–29	103	30
≥ 30	22	6
Maternal education		
None	46	13
Partial/complete primary	159	47
Partial secondary or more	137	40
Maternal literacy		
Illiterate/partially literate	100	29
Fully literate	242	71
Religion		
Muslim	262	77
Hindu	80	23
Wealth terciles		
Lower	123	36
Middle	117	34
Upper	102	30
Number of household members		
Up to five	125	36
Six to 10	153	45
More than 10	64	19

*N* = 342. Maternal and household characteristics were assessed during the baseline survey in 2015.

**Table 2:** Food and food hygiene characteristics of participant women and households in Sylhet, Bangladesh

Characteristics	Frequency	%
Food contamination		
Sample positive for *Escherichia coli*	156	46
Absent/low (< 10 CFU/g)	186	54
Medium (10–100 CFU/g)	62	18
High (> 100 CFU/g)	94	28
Household spot-checks		
At least one functional handwashing station	94	27
Water present	337	99
Soap present	102	30
Access to latrine (*N* = 341)*		
None/open defecation	6	2
Unimproved	24	7
Limited	138	40
At least basic	173	51
Kitchen area clean	64	19
Food preparation area clean	39	18
Reported handwashing at critical time points		
Before food preparation	103	30
Before child feeding	84	25
After defecation	204	60
After cleaning the child	79	23
After handling animal/child feces	108	32
All critical time points for handwashing mentioned	14	4
Other reported food hygiene behaviors		
Clean feeding utensils	195	57
Safe storage of food and water	35	10
Food prepared fresh or reheated properly	281	82
Observed handwashing at critical time points		
Before food preparation (*N* = 280)		
With soap	1	0.4
Any	16	6
Before child feeding (*N* = 301)		
With soap	7	2
Any	12	4
After defecation (*n* = 19)		
With soap	1	5
Any	4	21
After cleaning the child (*N* = 109)		
With soap	9	8
Any	18	16
After handling animal feces (*N* = 36)		
With soap	5	14
Any	13	36
Other observed food hygiene behaviors		
Clean feeding utensils (*N* = 273)	59	22
Safe storage of food and water	87	25
Food cooked fresh	124	36
Food not fresh but reheated	33	10
Food not fresh and not reheated	185	54
Food characteristics		
Type of food		
Porridge	33	10
Plain rice	38	11
Plain rice with pulses/vegetables/egg†	135	40
Plain rice dish pulses/vegetables/egg and fish/meat‡	69	20
Khichuri§	60	18
Other	6	2
Storage time of food		
< 2 hours	148	43
2–4 hours	63	19
4–8 hours	89	26
> 8 hours	42	12
	Mean	SD
Temperature of food storage area (in °C)	31.0	1.8
Humidity of food storage area (in %)	84	5.6

CFU/g = colony forming units per gram of food. *N* = 342; indicated in parentheses if this differed. Food and food hygiene characteristics were assessed in 2018.

* No information on excreta disposal, therefore it was not possible to distinguish between basic and safely managed facility.

† Plain rice served with pulses and/or vegetables and/or eggs.

‡ Plain rice served with pulses and/or vegetables and/or eggs and fish or meat.

§ Traditional dish of cooked rice and pulses.

Reported handwashing behavior was low: 30% of women said they washed their hands with soap before food preparation and 25% before child feeding. Only 4% of women mentioned handwashing with soap at all five critical times. Reported practice of other food hygiene behaviors ranged from 10% of women reportedly practicing safe storage of food and water, to 57% reportedly using clean feeding utensils and 82% of women reporting to either cooking food fresh or reheating stored food before feeding it to the child (Table 2).

Household observations and spot-checks showed that 27% of households had a functional handwashing station. This was mainly due to the low availability of soap at the handwashing stations (only in ∼30% of households, Table 2), not due to a lack of water. Less than 20% of households had a visibly clean kitchen or food preparation area, 22% used clean utensils for child feeding during observation, and 25% had stored food and water safely. Furthermore, most food storage areas had environments that favored bacterial growth (mean temperature of 31°C and 84% humidity). More than a third of food was freshly prepared, and 10% of food was reheated (8% was > 70°C at time of serving), whereas in more than half of the observations, food was not fresh and not reheated (Table 2). Potential occasions for handwashing at all five critical time points could not be observed in all households during the 3-hour observation period. When critical events were observed, good handwashing practice was rarely seen. For example, of all households with observed food preparation events, only one (0.4%) practiced good handwashing with soap before food preparation and only seven (2%) before child feeding (Table 2). Because there were few overall observations in some handwashing categories (e.g., after defecation) and very few positive observations in all handwashing categories, observed handwashing at critical times could not be included in subsequent analyses.

### Association of food hygiene behaviors and *E. coli* contamination.

In bivariable analysis, reported handwashing at critical times, reported use of clean feeding utensils, and reported safe storage of food and water were associated with reduced odds of food contamination, whereas reported fresh preparation or proper reheating of food showed a rather weak association with food contamination (Supplemental Table 1). From the observed food hygiene behaviors and household spot-checks, presence of a functional handwashing station, cleanliness of feeding utensils, and fresh preparation of food or reheating of stored food were identified as potential determinants of food contamination (Supplemental Table 1). There was only weak evidence that cleanliness of the kitchen area or safe storage of food and water were associated with food contamination (Supplemental Table 1). In addition, dishes prepared with plain rice and food stored for more than 2 hours were more likely to show increased food contamination, as was higher temperature of the food storage area (Supplemental Table 1).

**Figure 1.**
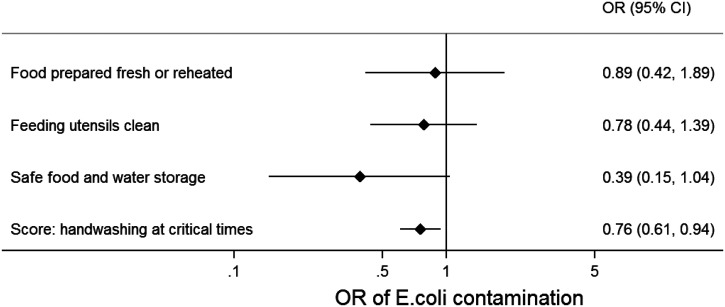
Association of reported maternal food hygiene behaviors and complementary food contamination in Sylhet, Bangladesh. Odds ratios (OR) of *Escherichia coli* contamination in complementary food samples (*N* = 342), with 95% confidence intervals from multilevel multivariable regression model with reported maternal food hygiene behaviors, adjusted for temperature and humidity of the food storage area, food type, food storage time, household wealth, maternal literacy, and intervention allocation, with settlement random effects.

In the multivariable model of reported food hygiene behaviors and *E. coli* contamination, we found that reported handwashing practice score was associated with reduced contamination of complementary food (adjusted odds ratio [OR]: 0.8, 95% confidence interval [CI]: 0.6–0.9), as was reported safe storage of food and water, although evidence for this association was weak (adjusted OR: 0.4, 95% CI: 0.1–1.0; Figure 1 and Supplemental Table 2). Accordingly, predicted probabilities of *E. coli* contamination were lower when handwashing was reported at more occasions and when food and water were reported to be stored safely (Figure 3A).

**Figure 2.**
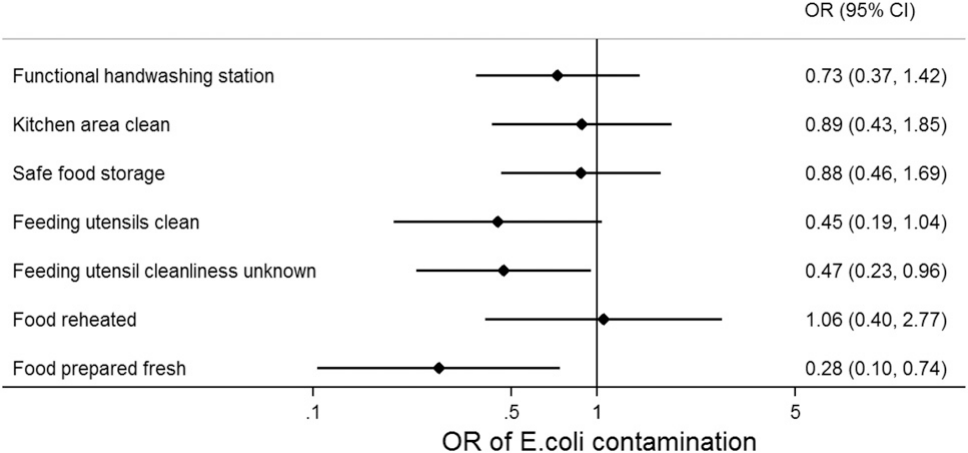
Association of observed food hygiene behaviors and spot‐checks and complementary food contamination in Sylhet, Bangladesh. Odds ratios (OR) of *Escherichia coli* contamination in complementary food samples (*N* = 342), with 95% confidence intervals, from multilevel multivariable regression model with observed food hygiene behaviors and spot‐checks, adjusted for temperature and humidity of the food storage area, food type, food storage time, household wealth, maternal literacy, and intervention allocation, with settlement random effects.

**Figure 3.**
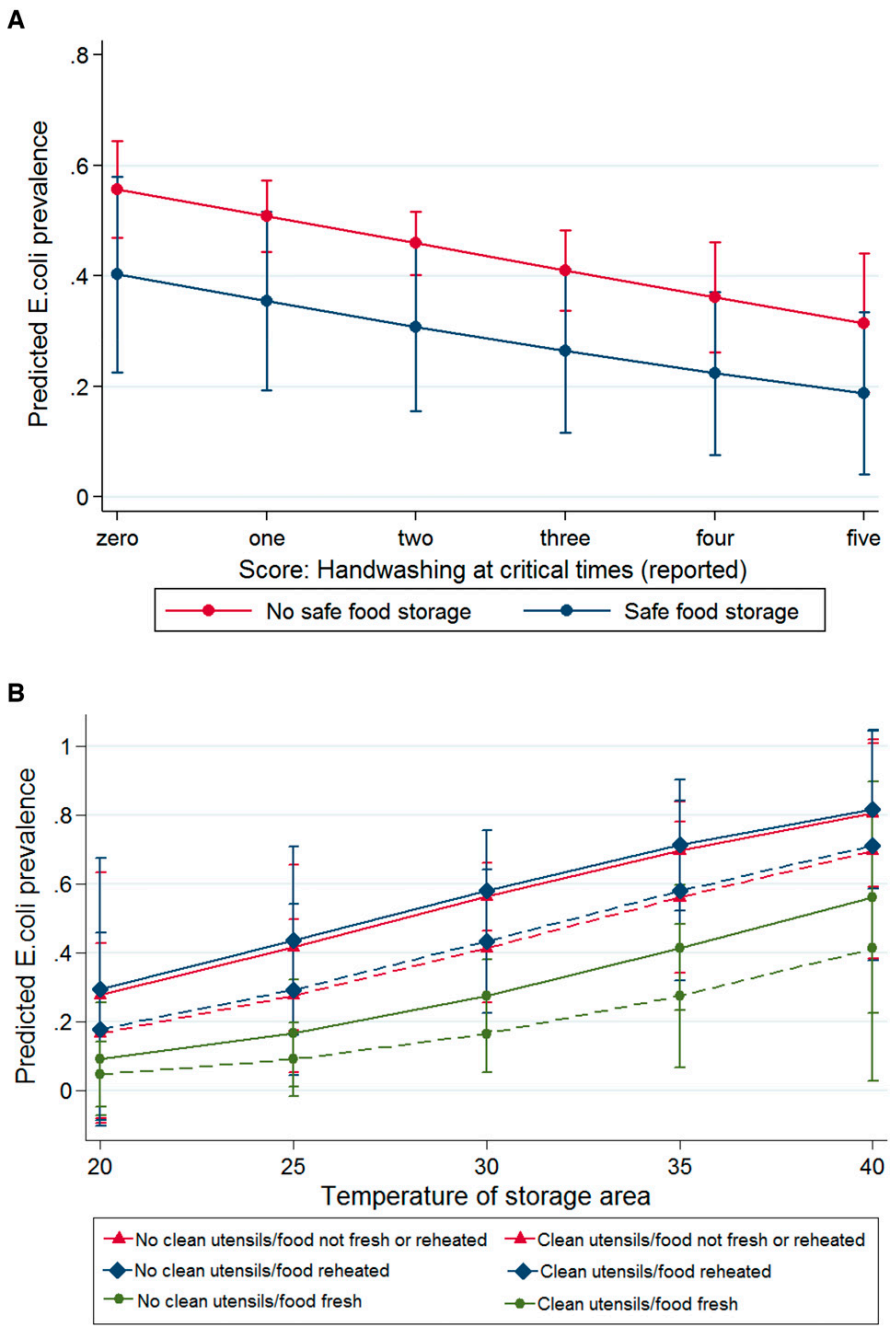
Predicted probabilities of *Escherichia coli* complementary food contamination in Sylhet, Bangladesh. Marginal probabilities of *E. coli* contamination and (**A**) reported handwashing at critical times by safe storage, (**B**) observed utensils and food reheating status by storage temperature. Results are from multilevel multivariable regression models, adjusted for temperature and humidity of the food storage area, food type, food storage time, household wealth, maternal literacy, and intervention allocation, with settlement random effects. Dotted lines: Clean utensils. *N* = 342. This figure appears in color at www.ajtmh.org.

In the multivariable model of observed food hygiene behaviors and *E. coli* contamination, fresh preparation of food was strongly associated with lower odds of complementary food contamination (adjusted OR: 0.3, 95% CI: 0.1–0.7). There was also an association between the cleanliness of feeding utensils and food contamination, although evidence for this association was weak (adjusted OR: 0.4, 95% CI: 0.2–1.0). Although presence of a functional handwashing station as well as reheating of stored food were associated with food contamination in bivariable analyses (Supplemental Table 1), in the adjusted multivariable model, there was little evidence for an association (adjusted OR [handwashing station]: 0.7, 95% CI: 0.4–1.4, and adjusted OR [reheating]: 1.1, 95% CI: 0.4–2.8, Figure 2 and Supplemental Table 3). Even when considering the stricter definition of reheating (food temperature > 70°C at the time of serving), there was no evidence for an association with reduced food contamination (adjusted OR: 1.2, 95% CI: 0.4–3.4). Accordingly, predicted probabilities of *E. coli* contamination were lowest in freshly prepared food served with clean feeding utensils (Figure 3B).

Because there was no evidence from multivariable models that reheated stored foods contained fewer *E. coli*–positive samples than nonreheated stored foods, we investigated whether reheating had an effect on reducing the degree of contamination in *E. coli*–positive samples. There was no evidence that reheating reduced *E. coli* counts (–0.3 log CFU/g food, 95% CI: –0.9 to 0.4), whereas freshly prepared food had lower *E. coli* counts compared with stored and not reheated food (–0.7 log CFU/g food, 95% CI: –1.4 to 0.03; Supplemental Table 4).

**Figure 4.**
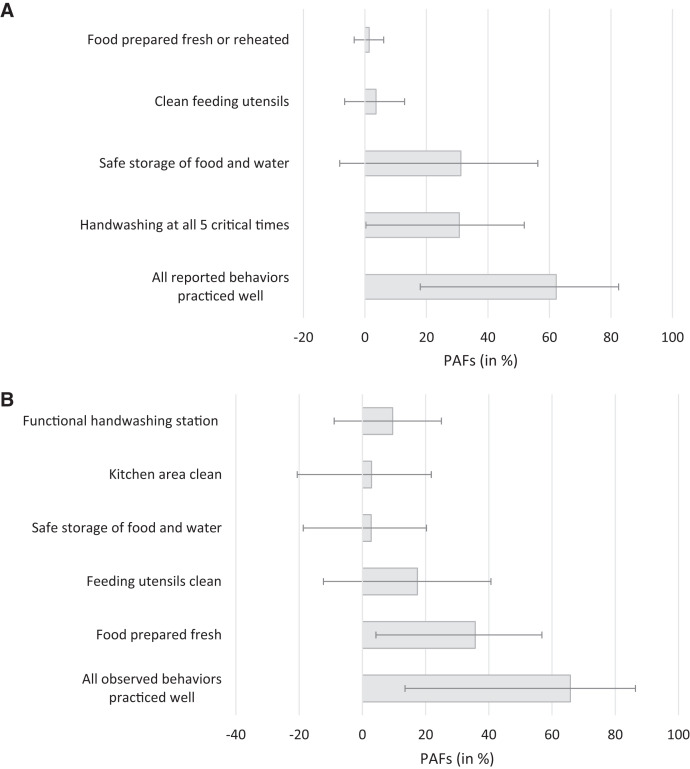
Population attributable fractions of food hygiene behaviors in Sylhet, Bangladesh. Population attributable fractions (PAF) were calculated following multivariable logistic regression with robust standard errors for A) reported food hygiene behaviors and B) observed food hygiene behaviors and spot‐checks in households (*N* = 342). PAFs are shown in % with 95% confidence intervals.

We calculated population attributable fractions to estimate the potential reduction of food contamination if the entire study population practiced good food hygiene behaviors. If all women practiced good handwashing at all critical times, food contamination could be reduced by nearly a third (PAF: 31%, 95% CI: 0.3–52%, Figure 4A and Supplemental Table 5). Food contamination could also be reduced by approximately a third if food was always prepared freshly by all households in our population (PAF: 36%, 95% CI: 4–57%; Figure 4B and Supplemental Table 5). For all other food hygiene characteristics, there was no strong evidence that perfect practice of a single behavior would lead to a substantial reduction of food contamination in this population (Figure 4 and Supplemental Table 5). Good practice of all five observed food hygiene behaviors by all households in this population was estimated to be able of reducing food contamination by two-thirds (PAF: 66%, 95% CI: 14–86%), and good practice of all four reported food hygiene behaviors by a similar amount (PAF: 62%, 95% CI: 18–83%; Figure 4 and Supplemental Table 5).

## DISCUSSION

We found a high prevalence of microbial contamination in complementary foods in our study population in rural Sylhet, Bangladesh, with nearly half of food samples positive for *E. coli*, which is comparable to levels observed by previous studies in Bangladesh and other low-income settings.^8^^,^^12^^,^^13^^,^^32^^–^^34^ Maternal practice of safe food hygiene behaviors was uncommon, and particularly handwashing with soap was rarely done. Reported handwashing with soap at critical times and fresh preparation of food before feeding were strongly associated with reduced food contamination, and there was no evidence that reheating of stored food or safe food storage were associated with reduced contamination. Evidence for an association of clean feeding utensils with reduced food contamination was also weak.

Our results on handwashing are in line with previous studies in low-income settings reporting high contamination of mothers’ and children’s hands with fecal bacteria,^33^^,^^35^^,^^36^ and an increased risk of contamination of food and food preparation/serving utensils when the food handler had bacterially contaminated hands.^37^^,^^38^ Child feeding is mainly done by hand in our study population, thus hand cleanliness is a good measure to avoid direct contamination of foods. Accordingly, we found that handwashing—if practiced by all women of our study population—would be able to reduce food contamination substantially. However, similar to prior observations in rural Bangladesh and rural Kenya,^38^^–^^40^ handwashing was rather uncommon in our study households; only 4% of mothers reported to wash hands at all five critical time points, and observed handwashing practice was even lower than reported behavior. Women likely overreport handwashing practice in face-to-face interviews because they want to meet expectations regarding hygiene behavior, resulting in social desirability bias.^41^^,^^42^ This means that the effect of handwashing on food contamination is likely underestimated in an analysis of reported behavior. Unfortunately, we were unable to include observed handwashing practice in regression analyses because we observed only a few overall events in some handwashing categories and no more than nine events of handwashing with soap in any handwashing category. Poor handwashing practice might be partially due to the low availability of functional handwashing stations in the study households, mainly caused by a lack of soap. In addition, handwashing stations are often not located near the kitchen or even outside in the yard. This lack of functional infrastructure has been previously described as a barrier to better handwashing practice in Bangladesh and Kenya.^23^^,^^39^

Safe practice of food and water storage showed no clear association with a reduction of food contamination in our study. This is in line with two studies in Bangladesh and Thailand^11^^,^^43^ but contrasts with other studies in Bangladesh and other low-income settings that found an association of food contamination with food storage practices.^12^^,^^13^^,^^19^^,^^34^ In addition to safe storage of food, storage time and temperature of the storage area have shown to be associated with food contamination in previous studies.^8^^,^^9^^,^^11^^–^^13^^,^^15^^,^^21^^,^^34^ In our study households, temperature of the storage area was generally high (between 27 and 37°C) and refrigeration was uncommon, so storage of leftover food at high ambient temperatures is likely. Similar to previous studies, we saw an increased risk of bacterial food contamination even after 4 hours of storage time.^8^^,^^11^ Thus, storage of leftover food is likely to pose a risk of food contamination in our study setting, even with good storage practices (in terms of keeping food covered and elevated from the ground), due to high storage temperatures and long storage times. Unfortunately, the high storage temperature is beyond the control of most households because refrigerators are not affordable for most families and difficult to run because of the unreliable power supply.

In line with this, fresh preparation of food showed the highest association with reduced complementary food contamination. If practiced universally, it would reduce the proportion of contaminated foods by more than a third in our study population. However, fresh preparation of food several times a day is rather uncommon in this setting. During structured observations, only about one-third of women had prepared food fresh before feeding the child, and data collected during FAARM routine surveillance showed that most households cook twice a day, while children are fed, on average, four times a day. Rice in particular is often a meal-to-meal leftover, which is cooked only once or twice a day but eaten at all meals. This could explain the high association between dishes with plain rice and food contamination compared with other foods. Promoting fresh food preparation at all times when a small child is fed is not feasible because lighting the stove and cooking several times a day is both time-consuming and costly in terms of fuel. A safe alternative to preparing food fresh could be proper reheating (> 70°C) of stored food before feeding, which is advocated as food safety measure by food safety offices and the WHO^44^ and has been shown to effectively reduce food contamination in different food types.^17^^,^^45^ Surprisingly, we found no evidence that reheating of stored food was associated with a reduction of food contamination in our setting. This lack of an association could be due to improper reheating practice in our study households (reheating < 70°C). However, even if we considered only samples as reheated that were proven to be reheated to > 70°C (temperature measured at point of serving), we failed to see an association with reduced food contamination (OR: 1.2, 95% CI: 0.4–3.4). Depending on the amount and type of food reheated, uneven temperature distribution or insufficient reheating time could have been responsible for the remaining bacterial contamination. Another possible explanation lies in the choice of outcome variable in the multivariable regression models, where we included bacterial contamination as a binary indicator (presence or absence of bacterial contamination), without considering the information about the degree of contamination. If reheating led to a reduction of *E. coli* counts but not to a complete absence, this effect is not picked up by the model. However, even when looking at the degree of contamination in the *E. coli*–positive samples, there was also no evidence that reheating reduced *E. coli* counts. Therefore, it is likely that reheating was not practiced well enough in our study population to substantially reduce the level of food contamination.

In line with other studies in Mozambique and Kenya,^12^^,^^46^ we found no or only weak evidence of an association between food contamination and cleanliness of the food preparation area or of feeding utensils. Therefore, apart from handwashing at critical times and fresh preparation of food, most food hygiene behaviors showed a weak association with reduced food contamination in our study population. The predicted probabilities of food contamination for a combination of different food hygiene behaviors might suggest that practicing a variety of food hygiene behaviors at the same time could lead to a more substantial reduction in food contamination. However, it remains to be shown whether the uptake of multiple food hygiene behaviors at once is feasible in practice. More detailed analyses of food hygiene practices among the women in the FAARM intervention arm (Sobhan et al., manuscript in preparation) will tell whether the uptake of multiple behaviors was seen and if this led to a reduction in food contamination.

### Strengths and limitations.

A strength of this study is the estimation of food hygiene behaviors not only through reported behavior but also through environmental spot-checks and observed food hygiene practices. Environmental spot-checks and observations are less prone to social desirability bias than maternal reporting of hygiene behaviors. However, a certain level of social desirability bias might still be present due to women reacting differently in the presence of an observer, meaning that the mothers or caretakers could have changed their behavior as a result of being observed by another person.^41^^,^^47^ We tried to minimize the risk of observation bias by not communicating the actual focus of the observation to the household. Maternal reports are also important because they provide information on those hygiene practices that we were unable to observe frequently enough during the structured observations (e.g., handwashing after defecation).

The study also has some limitations. We only sampled complementary foods in a subset of FAARM households (*N* = 342), those who had a child in the first year of complementary feeding. Due to the limited sample size, some subgroups in our analyses contained few data points, making estimates less precise than would be desirable. Also, we used the detection of fecal indicator bacteria as a proxy for the presence of pathogens in food. This might lead to an over- or underestimation of the presence of pathogenic bacteria because presence of fecal indicator bacteria does not necessarily imply the presence of pathogens and vice versa. However, the presence of fecal indicator bacteria indicates fecal contamination of food samples, which poses a health risk.^48^ Another limitation of the study is its cross-sectional design. FAARM is a cluster-randomized controlled trial, whereas for the present observational study, we combined the intervention and control groups of the trial to increase power for analyzing the association of food contamination with reported and observed food hygiene practices. Although we tried to control for potential confounding by adjusting for intervention arm and known household and woman characteristics, our results might be still affected by residual, unmeasured confounding—for example, the presence of animals in the household or the degree of environmental contamination (e.g., chicken feces or other fecal contamination). In addition, household data were collected in 2015, and certain characteristics (such as household wealth) could have changed in some households between then and food hygiene data collection in 2018–2019. This could have led to nondifferential misclassification of confounders, which could imply incomplete control for confounding. In addition, food sampling and assessment of food hygiene practices were only performed once per household, and the level of food contamination and the food hygiene practice observed or reported that day does not necessarily represent usual household practice; thus, we might over- or underestimate the effect of a single food hygiene behavior.

### Conclusion.

In this study, we identified key food hygiene behaviors associated with a reduction of complementary food contamination in a rural setting in Bangladesh. On the basis of these findings, improving hand hygiene and encouraging preparation of fresh food for young children as often as possible might be the most promising food hygiene messages to promote in this setting to achieve a reduction in complementary food contamination. However, advocating behavior change would need to go hand in hand with the improvement of necessary infrastructure, such as designing and establishing a low-cost, locally acceptable, and easily maintainable washing station with running water, sink, and proper drainage in or near the kitchen to enable consistent practice of handwashing and utensil cleaning.

Although data were only collected from parts of two rural subdistricts in Sylhet division, we believe that the household environment and food hygiene practices resemble typical rural areas of Bangladesh. It is thus likely that our results are relevant for households of similar demographic characteristics across the country—and possibly other countries in South Asia—and could therefore inform intervention designs. Future studies will show whether a successful reduction in complementary food contamination also has an actual impact on health—for example, by reducing child diarrhea or intestinal inflammation.

## Supplemental files


Supplemental materials
