# Supplementary material for: Key Food Hygiene Behaviors to Reduce Microbial Contamination of Complementary Foods in Rural Bangladesh
Source: Am J Trop Med Hyg. 2022 Jul 25;107(3):709–19. doi: 10.4269/ajtmh.21-0269 (PMC9490651; doi:10.4269/ajtmh.21-0269)
Supplement: Supplementary file 1 [file tpmd210269.SD1.pdf]

Supplemental Table 1. Association of single food hygiene behaviors and *E. coli* contamination of complementary foods in Sylhet, Bangladesh.

		Sample positive for *E. coli*			
**Food hygiene characteristics**		freq. (%)	OR	95% CI	p-value
Household spot checks					
At least one functional handwashing station	*No*	129 (52%)	0.4	0.2 - 0.7	< 0.001
	Yes	27 (29%)			
Kitchen area clean	*No*	134 (48%)	0.5	0.3 - 1.05	0.07
	Yes	22 (34%)			
Food preparation area clean	*No*	79 (45%)	0.8	0.3 - 2.0	0.64
	Yes	16 (41%)			
Reported handwashing at critical times					
Before food preparation	*No*	120 (50%)	0.5	0.3 - 0.9	0.02
	Yes	36 (35%)			
Before child feeding	*No*	130 (50%)	0.4	0.2 - 0.7	0.002
	Yes	26 (31%)			
After defecation	*No*	74 (54%)	0.6	0.3 - 0.97	0.04
	Yes	82 (40%)			
After cleaning the child's bottom	*No*	129 (49%)	0.6	0.3 - 1.05	0.07
	Yes	27 (34%)			
After handling animal/child feces	*No*	119 (51%)	0.4	0.2 - 0.7	0.003
	Yes	37 (34%)			
Score: handwashing at critical times (per additional time point mentioned)			0.7	0.6 - 0.8	< 0.001
Other reported food hygiene behaviors					
Feeding utensils clean	*No*	79 (54%)	0.5	0.3 - 0.9	0.01
	Yes	77 (40%)			

Safe storage of food and water	*No*	146 (48%)	0.4	0.2 - 0.9	0.04
	Yes	10 (29%)			
Food prepared fresh or reheated properly	*No*	35 (57%)	0.6	0.3 - 1.2	0.19
	Yes	121 (43%)			
Observed food hygiene behaviors					
Clean feeding utensils	*No*	111 (52%)	*Ref.*		
	Yes	16 (27%)	0.3	0.2 - 0.7	0.002
	Unknown	29 (42%)	0.7	0.4 – 1.3	0.25
Safe storage of food and water	*No*	121 (48%)	0.7	0.4 - 1.2	0.18
	Yes	35 (40%)			
Food prepared fresh		24 (19%)	0.1	0.07 - 0.2	< 0.001
Food not fresh but reheated		14 (42%)	0.4	0.2 - 0.9	0.04
Food not fresh and not reheated		118 (64%)	*Ref.*		
Food characteristics					
Type of food					
Porridge		6 (18%)	*Ref.*		
Plain rice		24 (63%)	8.9	2.7 – 29.6	< 0.001
Plain rice with pulses/vegetables/egg[#]		74 (55%)	6.7	2.4 – 19.0	< 0.001
Plain rice with pulses/vegetables/egg and fish/meat[##]		44 (64%)	8.7	2.9 – 26.1	< 0.001
Khichuri[###]		6 (10%)	0.5	0.1 – 1.8	0.29
Other		2 (33%)	1.9	0.2 – 15.0	0.55
Storage time of food					
< 2 hours		39 (26%)	*Ref.*		
2 - 4 hours		30 (48%)	2.5	1.2 – 4.9	0.01
4 - 8 hours		61 (69%)	7.1	3.6 – 14.0	< 0.001
> 8 hours		26 (62%)	6.4	2.7 – 15.3	< 0.001
Temperature of food storage area (per °C)			1.2	1.03 – 1.4	0.02
Humidity of food storage area (per %)			0.98	0.9 – 1.03	0.44

Total N=342; a child feeding event could not be observed in 69 households, in these households, food samples were taken after the observation period and status of feeding utensils was grouped as unknown; freq.: frequency, OR: odds ratio, CI: confidence interval; from multilevel regression models, adjusting only for settlement random effects. [#]Plain rice served with pulses and/or vegetables and/or eggs; [##] Plain rice served with pulses and/or vegetables and/or eggs and fish or meat; [###] Khichuri: traditional dish of jointly cooked rice and pulses.

Supplemental Table 2. Adjusted associations of *reported* food hygiene behaviors and *E. coli* contamination of complementary foods in Sylhet, Bangladesh

**Reported food hygiene behaviors**	OR	95% CI	p-value
Score: handwashing at critical times	0.8	0.6-0.9	0.01
Feeding utensils clean	0.8	0.4-1.4	0.44
Safe storage of food and water	0.4	0.1-1.0	0.06
Food prepared fresh or reheated	0.9	0.4-1.9	0.75
Type of food			
Porridge	*Ref.*		
Plain rice	6.4	1.7-23.7	0.006
Plain rice with pulses/vegetables/egg[#]	4.2	1.4-12.9	0.01
Plain rice with pulses/vegetables/egg and fish/meat[##]	4.0	1.2-13.1	0.02
Khichuri[###]	0.5	0.1-1.9	0.31
Other	1.6	0.2-14.5	0.67
Storage time of food			
< 2 hours	*Ref.*		
2 - 4 hours	1.7	0.8-3.7	0.21
4 - 8 hours	4.1	1.9-8.6	<0.001
> 8 hours	2.9	1.2-7.3	0.02
Temperature of food storage area (in °C)	1.1	0.95-1.4	0.16
Humidity of food storage area (in %)	1.0	0.94-1.1	0.93

Total N=342; OR: odds ratio, CI: confidence interval; from multivariable mixed effects logistic regression model adjusting for temperature and humidity of the food storage area, food type, day of food preparation (coefficients shown in table), as well as household wealth, maternal literacy, intervention allocation and settlement clustering using random effects. [#]Plain rice served with pulses and/or vegetables and/or eggs; [##] Plain rice served with pulses and/or vegetables and/or eggs and fish or meat; [###] Khichuri: traditional dish of jointly cooked rice and pulses.

Supplemental Table 3. Adjusted association of *observed* food hygiene behaviors and *E. coli* contamination of complementary foods in Sylhet, Bangladesh.

**Observed food hygiene behaviors and spot-checks**	OR	95% CI	p-value
Functional handwashing station present	0.7	0.4-1.4	0.4
Kitchen area clean	0.9	0.4-1.8	0.8
Feeding utensils clean	0.4	0.2-1.0	0.06
Feeding utensils not clean	*Ref.*		
Feeding utensils cleanliness unknown	0.5	0.2-0.96	0.04
Safe storage of food and water	0.9	0.5-1.7	0.7
Food prepared fresh	0.3	0.1-0.7	0.01
Food reheated	1.1	0.4-2.8	0.9
Food not fresh and not reheated	*Ref.*		
Type of food			
Porridge	*Ref.*		
Plain rice	3.7	0.98-13.6	0.05
Plain rice with pulses/vegetables/egg[#]	2.3	0.8-7.1	0.1
Plain rice with pulses/vegetables/egg and fish/meat[##]	2.3	0.7-7.7	0.2
Khichuri[###]	0.3	0.08-1.3	0.1
Other	1.1	0.1-10.8	0.9
Storage time of food			
< 2 hours	*Ref.*		
2 - 4 hours	0.8	0.3-2.2	0.7
4 - 8 hours	1.8	0.7-4.9	0.2
> 8 hours	1.2	0.4-3.8	0.7
Temperature of food storage area (in °C)	1.1	0.93-1.3	0.3
Humidity of food storage area (in %)	1.0	0.93-1.05	0.8

Total N=342, a child feeding event could not be observed in 69 households, in these households food samples were taken after the observation period and status of feeding utensils was grouped as unknown; OR: odds ratio, CI: confidence interval; from multivariable mixed effects logistic regression model adjusting for temperature and humidity of the food storage area, food type, day of food preparation (all shown above), household wealth, maternal literacy, intervention allocation and settlement clustering using random effects. [#]Plain rice served with pulses and/or vegetables and/or eggs; [##] Plain rice served with pulses and/or vegetables and/or eggs and fish or meat; [###] Khichuri: traditional dish of jointly cooked rice and pulses.

Supplemental Table 4. Association of *observed* food hygiene behaviors and *E. coli* count in *E. coli* positive complementary foods in Sylhet, Bangladesh.

**Observed food hygiene behaviors and spot-checks**	Coef.	95% CI	p-value
Functional handwashing station present	0.4	-0.1 - 0.8	0.1
Kitchen area clean	-0.3	-0.8 - 0.2	0.2
Feeding utensils clean	-0.3	-0.9 - 0.2	0.3
Feeding utensils not clean	*Ref.*		
Feeding utensil cleanliness unknown	-0.6	-1.0 - -0.1	0.01
Safe storage of food and water	-0.1	-0.5 - 0.3	0.6
Food prepared fresh	-0.7	-1.4 - 0.03	0.06
Food reheated	-0.3	-0.9 - 0.4	0.4
Food not fresh and not reheated	*Ref.*		
Type of food			
Porridge	*Ref.*		
Plain rice	0.3	-0.7 - 1.3	0.5
Plain rice with pulses/vegetables/egg[#]	0.5	-0.4 - 1.4	0.3
Plain rice with pulses/vegetables/egg and fish/meat[##]	0.5	-0.5 - 1.5	0.3
Khichuri[###]	0.9	-0.4 - 2.1	0.2
Other	2.1	0.3- 3.8	0.02
Storage time of food			
< 2 hours	*Ref.*		
2 - 4 hours	-0.6	-1.2 - 0.1	0.08
4 - 8 hours	0.1	-0.5 - 0.7	0.6
> 8 hours	-0.01	-0.7 - 0.7	0.97
Temperature of food storage area (in °C)	0.03	-0.09 - 0.1	0.7
Humidity of food storage area (in %)	-0.007	-0.04 - 0.03	0.7

Total N=156, the model includes only samples positive for *E. coli*; a child feeding event could not be observed in 29 households, in these households food samples were taken after the observation period and status of feeding utensils was grouped as unknown; Coef.: regression coefficient, changes are shown in log CFU/gram food; CI: confidence interval; from multivariable mixed effects linear regression model adjusting for temperature and humidity of the food storage area, food type, day of food preparation (all shown above), household wealth, maternal literacy, intervention allocation, and settlement clustering using random effects. [#]Plain rice served with pulses and/or vegetables and/or eggs; [##] Plain rice served with pulses and/or vegetables and/or eggs and fish or meat; [###] Khichuri: traditional dish of jointly cooked rice and pulses

Supplemental Table 5. Population attributable fractions of food hygiene characteristics in Sylhet, Bangladesh.

Food hygiene characteristics	PAF in % (95% CI)
Reported food hygiene behavior	
Score: handwashing at critical times	30.7 (0.3 – 51.8)
Feeding utensils clean	3.6 (-6.6 – 12.9)
Safe storage of food and water	31.2 (-8.2 – 56.2)
Food prepared fresh or reheated	1.4 (-3.5 – 6.1)
All four reported behaviors practiced	62.2 (18.0 – 82.5)
Observed food hygiene behaviors and spot-checks	
Functional handwashing station present	9.6 (-8.9 – 25.0)
Kitchen area clean	2.9 (-20.6 – 21.8)
Feeding utensils clean	17.4 (-12.3 – 40.7)
Safe storage of food and water	2.8 (-18.7 – 20.3)
Food prepared fresh	35.7 (4.3 – 56.8)
All observed behaviors practiced well	65.8 (13.5 – 86.4)

Total n=342; PAF: Population attributable fraction, CI: 95% confidence interval; PAFs were calculated following multivariable logistic regression with robust standard errors
